# Functional study of DAND5 variant in patients with Congenital Heart Disease and laterality defects

**DOI:** 10.1186/s12881-017-0444-1

**Published:** 2017-07-24

**Authors:** Fernando Cristo, José M. Inácio, Salomé de Almeida, Patrícia Mendes, Duarte Saraiva Martins, José Maio, Rui Anjos, José A. Belo

**Affiliations:** 1 Stem Cells and Development Laboratory, CEDOC, NOVA Medical School / Faculdade de Ciências Médicas, Universidade Nova de Lisboa, Lisboa, Portugal; 2 Center for Biomedical Research (CBMR), Universidade do Algarve, Faro, Portugal; 3 Biomedical Sciences, Universidade do Algarve, Faro, Portugal; 4 Regenerative Medicine Program, Biomedical and Medicine Sciences Department, Universidade do Algarve, Faro, Portugal; 5 Medical Genetics Service, Centro Hospitalar Lisboa Central (CHLC), EPE, Lisboa, Portugal; 6 Departamento Materno-Infantil, Centro Hospitalar do Algarve, EPE, Faro, Portugal; 7 Hospital de Santa Cruz, Centro Hospitalar Lisboa Ocidental, Lisboa, Portugal; 8 NOVA Medical School | Faculdade de Ciências Médicas, Universidade Nova de Lisboa, Edifício CEDOC II, Rua Câmara Pestana n.° 6, 1150-082 Lisboa, Portugal

**Keywords:** DAND5, Congenital Heart Diseases, laterality defects, Nodal signaling, allelic variation

## Abstract

**Background:**

Perturbations on the Left-Right axis establishment lead to laterality defects, with frequently associated Congenital Heart Diseases (CHDs). Indeed, in the last decade, it has been reported that the etiology of isolated cases of CHDs or cases of laterality defects with associated CHDs is linked with variants of genes involved in the Nodal signaling pathway.

**Methods:**

With this in mind, we analyzed a cohort of 38 unrelated patients with Congenital Heart Defects that can arise from initial perturbations in the formation of the Left-Right axis and 40 unrelated ethnically matched healthy individuals as a control population. Genomic DNA was extracted from buccal epithelial cells, and variants screening was performed by PCR and direct sequencing. A Nodal-dependent luciferase assay was conducted in order to determine the functional effect of the variant found.

**Results:**

In this work, we report two patients with a *DAND5* heterozygous non-synonymous variant (c.455G > A) in the functional domain of the DAND5 protein (p.R152H), a master regulator of Nodal signaling. Patient 1 presents left isomerism, ventricular septal defect with overriding aorta and pulmonary atresia, while patient 2 presents ventricular septal defect with overriding aorta, right ventricular hypertrophy and pulmonary atresia (a case of extreme tetralogy of Fallot phenotype). The functional analysis assay showed a significant decrease in the activity of this variant protein when compared to its wild-type counterpart.

**Conclusion:**

Altogether, our results provide new insight into the molecular mechanism of the laterality defects and related CHDs, priming for the first time DAND5 as one of multiple candidate determinants for CHDs in humans.

**Electronic supplementary material:**

The online version of this article (doi:10.1186/s12881-017-0444-1) contains supplementary material, which is available to authorized users.

## Background

Heart morphogenesis is a complex process involving multiple cell types (cardiac and non-cardiac) thus requiring a precise control of all molecular and cellular mechanisms [1]. Subtle deviations in heart development lead to Congenital Heart Diseases (CHDs), which are the most prevalent form of birth defect (postnatal incidence of 0.8% of all newborns) and the leading non-infectious cause of death in the first year of life [2, 3]. The etiology of CHD is incompletely understood, thought to be multifactorial, involving multiple genetic and environmental factors, and the possible interactions between those factors [4]. Nevertheless, in the last years, CHDs are seen as disorders resulting from alterations in genes that control basic embryonic processes between the third and eighth week of gestation, when the majority of the cardiovascular structures develop and when the integrity of the left-right (LR) axis is established [5, 6]. In fact, in the majority of cases presenting laterality disorders, a complex heart malformation is also observed, suggesting that CHDs could be due to a laterality defect in the formation of the heart [7]. In others words, even in children that born with CHDs and that do not present others extracardiac laterality abnormalities, the etiology of these CHDs could arise from subtle faults in the formation of the left-right axis.

The molecular control of LR axis development is mainly achieved by the Nodal signaling pathway [8]. Studies in mice revealed that during gastrulation, Nodal, a growth factor from the TGF-ß family, is asymmetrically expressed in the node, and this expression is expanded and amplified in the left-lateral plate mesoderm (L-LPM), but inhibited in the right-lateral plate mesoderm (R-LPM) [9]. Nodal itself induces its intracellular signaling by binding to type I (ALK4 and ALK7) and type II (ActRIIa and ActRIIb) serine-threonine kinase receptors in the presence of one EGF-CFC (epidermal growth factor-Cripto-FRL1-Cryptic) family co-receptor, Cripto or Cryptic. This leads to the phosphorylation of regulatory Smads (Smad2 and Smad3), their association with Smad4, translocation into the nucleus, and to the interaction with the transcription factor FoxH1, which at the end activates the expression of Nodal target genes, in particular, *Pitx2* [9]. *Pitx2* is a transcription factor that regulates the fate of cells that will form the visceral organs, including the heart [8, 10]. The impairment of the Nodal signaling in the lateral plate mesoderm, by variants in the genes involved in the pathway such as *ActRIIB* (MIM #602730) [11, 12], *LEFTY 1* (MIM #603037) [13, 14], *LEFTY 2* (MIM #601877) [13, 14], *CRYPTIC*/*CFC1* (MIM #605194) [15, 16], *CRIPTO*/*TDGF1* (MIM #187395) [17, 18], *FOXH1* (MIM #603621) [19, 20], *NODAL* (MIM #601265) [21, 22] and *PITX2C* (MIM # 601542) [23, 24] had already been associated to laterality disorders and/or CHDs in humans. For example, the heterozygous variant p.G260R in NODAL was associated with transposition of the great arteries (TGA), atrial and ventricular septal defects, double outlet right ventricle (DORV) and/or heterotaxy [21]. On the other hand, the NODAL p.S60I variant has been associated with the tetralogy of Fallot (TOF) phenotype [22]. Additionally, several variations in Nodal co-receptor CFC1 were linked to TOF, TGA, DORV and/or laterality defects [15, 17, 25].

Recently, we reported that a DAN family member, the Nodal antagonist *Cerberus-like 2* (*Cerl2*) – *DAND5* in humans (MIM #609068; refseq - NM_152654.2) – controls Nodal signaling at the mouse node [26, 27], and the transmission of LR asymmetry information to the left-lateral plate mesoderm in a precise time window [28, 29]. Furthermore, the absence of *Cerl2* in *Cerl2* KO embryos leads to a range of laterality defects and/or cardiovascular malformations such as incomplete atrial and ventricular septation, TGA, DORV, randomized positioning of the cardiac apex, ventricular hypertrophy and/or heterotaxy of the abdominal organs, and a significant mortality rate within a few hours after birth is observed [30, 31].

Here, in order to advance the understanding of DAND5 in the genetic etiology of laterality disorders and associated CHDs, we conducted a genetic screening to identify *DAND5* allelic variants in children affected with CHDs and/or LR asymmetry defects. We identified a *DAND5* allelic variant in two patients, which was absent in the referral chromosomes from an ethnically matched control population that reduces the inhibitory activity of DAND5. We suggest that this variant might contribute as a risk allele for congenital heart disease and/or laterality defects.

## Methods

### Ethics

This study was conducted in accordance with the ethical principles of the revised Declaration of Helsinki. The research protocol was approved by the local institutional ethics committee of Hospital de Santa Cruz, Centro Hospitalar de Lisboa Ocidental, E.P.E., Hospital de Faro, Centro Hospitalar do Algarve, E.P.E., Hospital Sta. Marta, Centro Hospitalar de Lisboa Central, E.P.E. and NOVA Medical School. The Research Project was approved by the National Committee for Data Protection (Authorization N.° 8694/2016). Written informed consent was obtained from all guardians participants prior to the collection of the DNA.

### Clinical evaluation and inclusion criteria

The study sample included 38 Caucasian unrelated pediatric patients with congenital heart defects arising from possible left-right defects recruited from three Portuguese hospitals, Hospital de Santa Cruz, Centro Hospitalar de Lisboa Ocidental, E.P.E., Hospital de Faro, Centro Hospitalar do Algarve, E.P.E., Hospital Sta. Marta, Centro Hospitalar de Lisboa Central, E.P.E. The sample was obtained at the time of the appointment according to the convenience and availability of physicians in each hospital. A total of 40 ethnically matched unrelated individuals were recruited as control samples to screen for the identified variant in *DAND5*.

Patients were evaluated by individual and familial history, review of the medical records and individual anatomic descriptions of the patients’ defects were obtained by the review of echocardiogram, echocardiography, magnetic resonance imaging, angiography or direct view during cardiac surgery. Taking into account the phenotype of the *Cerl2* KO mice, in which 35% of the homozygous mutants died within the first 48 h after birth due to several cardiovascular malformations, including incomplete atrial and ventricular septation, conotruncal defects, ventricular hypertrophy, and/or to laterality defects like left isomerism and thoracic heterotaxy or situs inversus, the inclusion criteria required the presence of congenital heart malformations and/or laterality defects, namely left or right isomerism; heterotaxy; situs inversus; transposition of great arteries (TGA); double inlet left ventricle (DILV); double outlet right ventricle (DORV); tetralogy of Fallot or Fallot like phenotype; atrioventricular septal defects (AVSD); atrial septal defects (ASD); ventricular septal defects (VSD) and ventricular hypertrophy. The patients with known chromosomal abnormalities or syndromic cardiovascular defects, such as Down syndrome, Turner syndrome, Marfan syndrome, Di George syndrome, and Holt–Oram syndrome, were excluded from the study.

The clinical characteristics of the cohort of patients that participated in the study are summarized in Table 1.

**Table 1:** Clinical characteristics of the patients genotyped

Prevalence of different types of CHD	Patients (*n* = 38)
Atrial septal defect	2 (~5%)
Ventricular septal defect	11 (~29%)
Atrioventricular septal defects	8 (~21%)
Conotruncal defects	
Pulmonary atresia/stenosis	14 (~37%)
Tetralogy of Fallot	7 (~18%)
Transposition of the great arteries	8 (~21%)
Double inlet left ventricle	2 (~5%)
Double outlet right ventricle	1 (~3%)
Aortic coarctation	1 (~3%)
Overriding aorta	1 (~3%)
Left isomerism	4 (~11%)
Right isomerism	1 (~3%)
Dextrocardia	3 (~8%)
Other cardiac malformations	20 (~53%)
Extracardiac abnormalities	
Situs inversus totalis	1 (~3%)
Visceral situs inversus	1 (~3%)
Asplenia	1 (~3%)

Other cardiac malformations included right sided aortic arch, inferior vena cava interruption, univentricular heart, aortic stenosis, mitral valve stenosis, tricuspid atresia, hypoplastic right ventricle, hypoplastic left heart, major aortopulmonary collateral artery, persistent left superior vena cava, single ventricle, total anomalous pulmonary venous connection. Note: Almost all the patients have more than one type of CHD

### Genetic screening of *DAND5* gene

Genomic DNA from the buccal epithelial cells of all participants was extracted using the Isolate Genomic DNA mini kit (BIOLINE) according to the manufacturer’s instructions for DNA isolation from buccal swabs. The coding exons and flanking exon–intron boundaries of the *DAND5* gene were sequenced in 38 Caucasian unrelated patients with CHDs and/or laterality defects and in 40 ethnicity- and geographic-matched controls. The referential genomic DNA sequence of *DAND5* was derived from Ensembl (http://www.ensembl.org), accession number ENST00000317060. Primer-BLAST program (http://www.ncbi.nlm.nih.gov/tools/primer-blast/) was used to design the primer pairs utilized to amplify the coding regions and splice junction sites of *DAND5*, by polymerase chain reaction, as follows: 1Forward: 5′-GTCGACTGCTAGTGACCTTGAG-3′; 1Reverse: 5′-TCAGGTGGAGGATACAGGACTT-3′; 2Forward: 5′-GGAAGTGGACAGGTGATTATCC-3′; 2Reverse: 5′-CACGTCTTTCTTGGTCCATCTC-3′. The PCR was carried out using 10–30 ng of genomic DNA in a 25 μL reaction containing 1X Phusion HF Buffer (ThermoScientific), 0.5 μM of each primer, 0.2 mM dNTPs and 0.02 U/μL of Phusion High-Fidelity DNA polymerase (ThermoScientific). PCR cycling was performed on C1000 Thermal Cycler (Bio-Rad) using a denaturation cycle at 98 °C for 1 min followed by 35 cycles of denaturation at 98 °C for 15 s, annealing of primers for 30 s and extension at 72 °C for 30 s and a final extension step at 72 °C for 5 min. Both strands of each PCR product were sequenced with an ABI Prism 3130 automated genetic analyzer (Applied Biosystems) according to the protocols from the Sequencing Service of CCMAR (http://www.ccmar.ualg.pt/). The DNA sequences were analyzed by BioEdit Sequence Alignment Editor Software. The identified *DAND5* variant was confirmed by sequencing three independent PCR-generated amplicons from the same patient. Additionally, the identified sequence variation was queried in the single nucleotide polymorphism (SNP) database at NCBI (https://www.ncbi.nlm.nih.gov/), the human gene mutation (HGM) database (http://www.hgmd.org/), the 1000 Genome Project (1000 GP) database (http://www.1000genomes.org/), and the ExAC consortium (http://exac.broadinstitute.org/) to confirm its novelty.

Screening for alterations in other known genes associated to CHD / laterality was not conducted in our patients and control cohorts.

### Sequence alignment of DAND5 among vertebrate species

Conservation of the amino acid altered by the missense allelic variant was estimated by aligning the human DAND5 protein to chimpanzee, rat, mouse, xenopus and zebrafish using Clustal Omega program from Uniprot (http://www.uniprot.org/).

### Characterization of the causative potential of p.R152H variation

The potential effect of the p.R152H DAND5 variation was characterized by the software programs Polyphen − 2 (http://genetics.bwh.harvard.edu/pph2/) Mutation Taster (www.mutationtaster.org), PROVEAN (http://provean.jcvi.org/), and I- Mutant 3.0 (http://gpcr2.biocomp.unibo.it/cgi/predictors/I-Mutant3.0/I-Mutant3.0.cgi). The outcome of all tests was either a disease mutation or a harmless polymorphism. Polyphen-2 utilizes a combination of sequence, function, evolutionary conservation and structure based attributes and uses naive Bayesian classifier to determine the effect of an amino acid alteration. The output levels of damaging in the protein were classified as deleterious (≤ 0.5) and the benign level being classified as tolerated (≥ 0.51). Mutation Taster software’s analysis are based on protein structure/function and evolutionary conservation and employs a Bayes classifier to eventually predict the disease potential of an alteration. PROVEAN based analysis takes into account the alignment and measurement of similarity between variant sequence and protein sequence homolog, and the output classifies the alteration as deleterious (≤ −2.5) or neutral (≥ 2.51). I-Mutant 3.0 is a support vector machine-based tool that predicts the stability change of a mutated protein. The output prediction classifies the alteration in 3 classes: neutral alteration (− 0.5 ≤ DDG ≤ 0.5 kcal/mol), large decrease of stability (≤ − 0.5 kcal/mol) and large increase of stability (> 0.5 kcal/mol).

### Plasmids and site-directed mutagenesis

The encoding full-length human *DAND5* (IRCMp5012B019D) was purchased from Source BioScience LifeSciences, Nottingham, United Kingdom. The c.455G > A variant was generated by site-directed mutagenesis using the QuikChange kit (Stratagene, La Jolla, CA, USA) and the following pair of primer: Forward: 5′ GTATGCCTGCTC**A**CAAGCGTTGGG 3′; Reverse: 5’CCCAACGCTTG**T**GAGCAGGC 3′ according to the manufacturer’s instructions. The recombinant expression vectors *hNODAL-*pcDNA and *hCRIPTO-*pEF6/V5-His TOPO were kindly provided by Dr. Michael R Kuehn from Center for Cancer Research, National Cancer Institute, Bethesda, Maryland and the FoxH1 plasmid was provided by An Zwijsen from KU Leuven. The plasmids were subcloned into the expression vector pCS^2+^ vector to ensure the same backbone plasmid for each gene.

### Luciferase assay

Human embryonic kidney 293T cells were cultured in Dulbecco’s modified Eagle’s medium supplemented with 10% fetal calf serum and seeded overnight at 50–60% confluence in 96-well plates. Next day, the cells were transiently co-transfected with Lipofectamine 2000 (Lifetechnologies) and the following plasmids: pAR3-lux, that contains three copies of the activin response element (ARE) in the front of a luciferase reporter, which is specifically activated by nodal signaling; hDAND5; hDAND5 R152H; hNODAL; hCRIPTO, a Nodal co-receptor necessary for the proper activation of the Nodal signaling; hFoxH1, the major transcriptional transducer of nodal signaling; CMV-β-Gal plasmid as control, and various amounts of pCS^2+^ vector to maintain a constant amount of total DNA. Transfections were performed in triplicate and in three independent experiments with a total amount of 100 ng DNA per well. Twenty-four hours after transfection, luciferase activity was analyzed and the activities were normalized to b-galactosidase control.

### Western blot

Human embryonic kidney 293 T cells were transiently transfected using lipofectamine 3000 (Thermo Fisher Scientific) in Opti-MEM I reduced-serum medium (Thermo Fisher Scientific). Cell lysates and conditioned media were collected after 24 h, and protein expression was monitored by Western blotting using polyclonal goat anti-Cerl2 (R&D), and HRP- anti-goat (Sigma) antibodies. Proteins were visualized using ECL detection reagent (Bio-Rad).

### Statistical analysis

The data obtained from all analyses was statistically analyzed using GraphPad PRISM 5 software. Statistical differences were determined by 2–tailed, unpaired Student’s *t*-test. Probability values of *P* < 0.05 were considered significant.

## Results

**Fig. 1:**
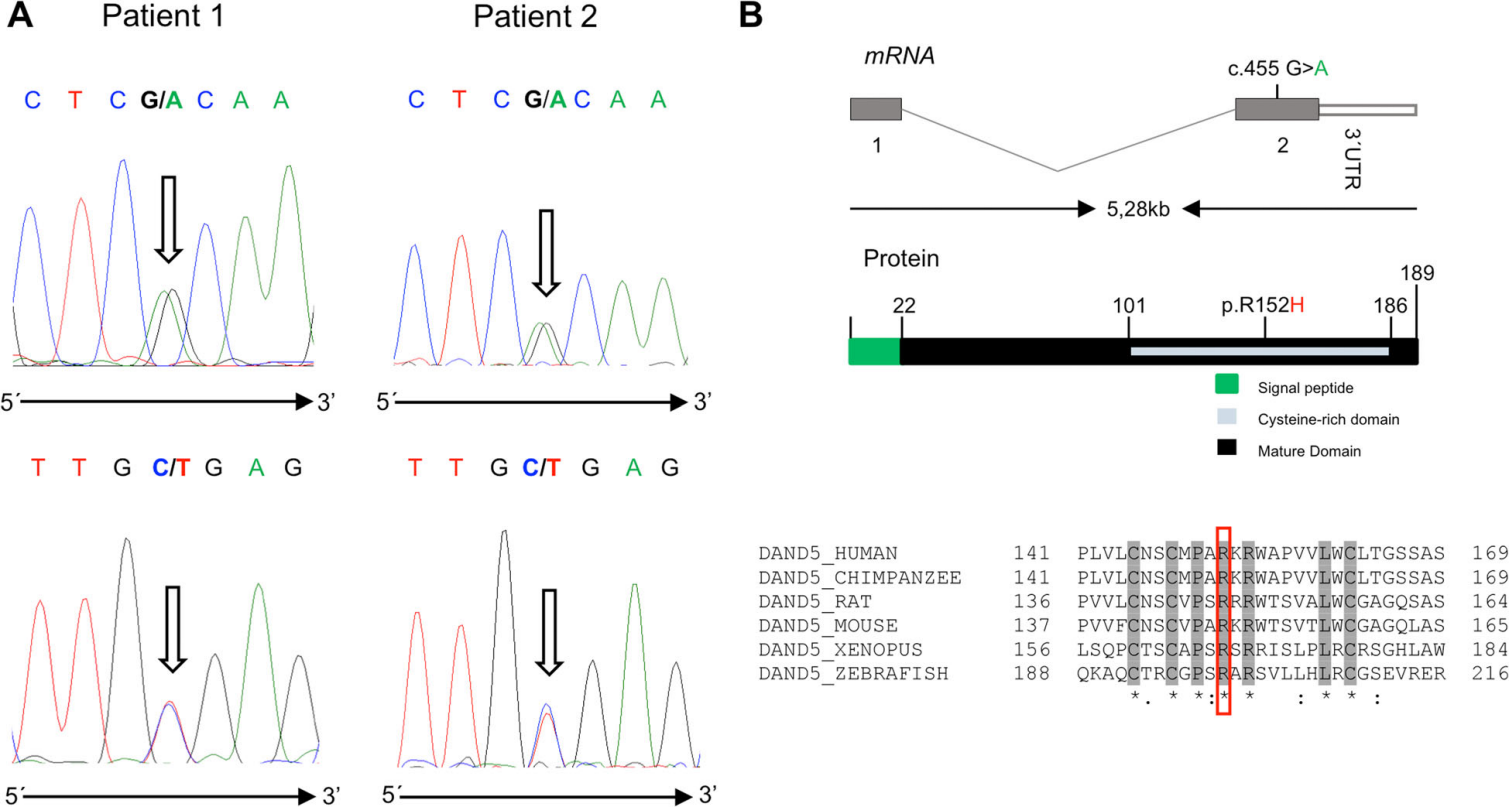
**a** Forward and reverse DNA sequence chromatograms of patient 1 and 2 showing the c.455G > A allelic variant (highlighted by *arrows*). **b** Schematic diagram of DAND5 structure with approximated localization of the variant identified in this study and cross-species sequence alignment of DAND5 showing the conservation of amino acid R152 (highlighted by *red box*)

**Fig. 2:**
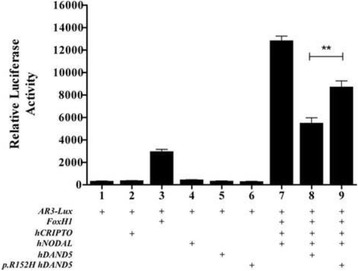
Functional analysis of DAND5 variant. The identified p.R152H variant was evaluated through a luciferase assay as readout of Nodal signaling. In this assay a luciferase reporter gene under the control of three activin-responsive elements promoter, pAR3-lux, which is transcriptionally activated by Nodal signaling, was used. The results, from triplicates of three independent experiments, showed that the p.R152H DAND5 variant leads to an increase of NODAL signaling when compared to WT DAND5 protein. The effect of the different genes altogether and the individual effect for each one are shown. Data are shown as mean values from three independent experiments, with the bars indicating the S.D. Asterisks: sample with a significant difference (*P* < 0.01*, t-*test) calculated from comparison with wild-type

DNA from 38 unrelated children with congenital heart defects arising from possible left-right defects and 40 ethnicity- and geographic-matched controls was direct sequenced for the detection of variants in the two exons of *DAND5* gene. Sequence analysis of our patient’s cohort revealed a non-synonymous c.455G > A heterozygous *DAND5* variant in two patients (Fig. 1a), not detected in in the control population. This alteration was also present has heterozygous in the apparently normal mother of patient 2. Unfortunately, the father of patient 2 and the parents of patient 1 were not available for sequencing. For validation, all exons were sequenced using forward and reverse internal primers flanking the missense variant. Clinically, patient 1 presented ventricular septal defect with overriding aorta, pulmonary atresia and left isomerism, while patient 2 presented overriding aorta, ventricular septal defect, right ventricular hypertrophy and pulmonary atresia (a case of extreme tetralogy of Fallot phenotype; Table 2). The c.455G > A variant in exon 2 of *DAND5* results in a substitution of an Arginine residue for a Histidine (p.R152H) localized in a highly conserved region of the cysteine-rich DAN domain (Fig. 1b). Interestingly, the DAND5 p.R152H variant had been already annotated in the NCBI (http://www.ncbi.nlm.nih.gov) and Ensemble (http://www.ensembl.org) databases as Single Nucleotide Variation (SNV - rs45513495), with a minor allele frequency of ~1% (low-frequency variant; Table 3) and described in ExAC database with an allele frequency of 0.01043 in all populations but never reported to be associated with disease. To decide whether the detected missense mutation is pathogenic or not, we use in silico analysis tools (PolyPhen2, Mutation Taster, PROVEAN and I-Mutant 3.0) and the results estimated differing functional effect of this variant in DAND5 (Table 4). Using the up-to-date PolyPhen-2 algorithm (http://genetics.bwh.harvard.edu/pph2/index.shtml), we observed that the DAND5 p.R152H variant had no effect on the DAND5 protein (classified as benign). Mutation Taster algorithm (www.mutationtaster.org) predicted that this variant is a polymorphism that might affect DAND5 protein features, namely alterations in the cysteine-rich domain. Moreover, PROVEAN software (http://provean.jcvi.org/) predicted a deleterious effect (score − 2.84) on the proper function of the DAND5 protein, and I-Mutant 3.0 predicted that the a.a. alteration will largely reduce the stability of the protein (a score of −1.28 a reliability index of 9; disease reliability index of 3). To clarify the function of this protein modification, we reproduced the p.R152H variant in an expression vector containing the human *DAND5* cDNA, and we evaluated the in vitro effect of p.R152H in the regulation of Nodal activity using a well-established Nodal-dependent luciferase assay as readout. This assay consists of a luciferase reporter gene under the control of three activin-responsive elements promoter, pAR3-lux, which is transcriptionally activated by Nodal signaling [21]. In addition, we used the human version of the main intervenient genes, *NODAL, CRYPTIC/CRIPTO, FOXH1*, all necessary for a reliable transduction of the levels of Nodal signaling in this type of assay. The results, from triplicates of three independent experiments, showed that the p.R152H DAND5 variant causes a significant reduction in the normal NODAL-inhibitory activity of DAND5 (Fig. 2). Moreover, a dose-response at three different concentrations for the wild-type vs. mutated protein confirms the reduced NODAL-inhibitory activity of DAND5 (Additional file 1: Figure S1 and Additional file 2: Figure S2, being the two forms equally produced upon transfection, Additional file 3: Figure S3).

**Table 2:** Clinical and molecular findings of patient 1 and 2

	Genotype	Protein alteration	Phenotype
Proband 1	*DAND5* c.455GA	DAND5 p.R152H	Left isomerism; VSD with overriding aorta; Pulmonary atresia
Proband 2	*DAND5* c.455GA	DAND5 p.R152H	Tetralogy of Fallot; Pulmonary atresia

VSD – ventricular septal defect

Wild type DAND5 refseq. - NM_152654.2

**Table 3:** *DAND5* c.455 G > A allele frequency according to ExAC genome project

Gene	Variant	Variant rs number
*DAND5*	c.455 G > A / p.R152H	45513495
Population	Allele frequency (%)	Number of homozygous / heterozygous
European (Finnish)	2,4	2 / 155
European (Non-Finnish)	1,5	14 / 990
All populations	1	16 / 1234

**Table 4:** In silico protein prediction effect of DAND5 p.Arg152His alteration

	Score	Prediction effect
PolyPhen-2	0,120	Benign
Mutation Taster	Polymorphism	Might affect protein features
PROVEAN	-2,84 (cutoff = −2,5)	Deleterious
I-Mutant 3.0	−1,28 Kcal/mol	Reduced stability of the protein and related to disease

## Discussion

The DNA sequencing analysis of our 38 unrelated children and 40 control cohorts, revealed two patients, patient 1 and patient 2, displaying the same variant in *DAND5* gene, not detected in the control population. One of the patients presented ventricular septal defect with overriding aorta, pulmonary atresia and left isomerism, while the other presented overriding aorta, ventricular septal defect, right ventricular hypertrophy and pulmonary atresia (a case of extreme tetralogy of Fallot phenotype). The phenotypically normal mother of patient 2 also presented heterozygosity the *DAND5* c.455G > A variant, suggesting incomplete penetrance. No others variants were detected neither in the *DAND5* gene of the 38 patients nor control population. This suggests that other variants in *DAND5* might have a more severe or lethal phenotype, or that due to its small length *DAND5* has less probability to randomly gain a new alteration.

The functional effect of the variant, which leads to a substitution of an arginine for a histidine in the position 152 of the highly conserved cysteine-rich DAN domain, was inconclusive in the predicted analysis performed with four different prediction algorithms. Nevertheless, these algorithms have only an accuracy of 65–80% in the prediction of known disease missense variants, being even less accurate when examining missense variants with milder effect [32]. Taking this in attention, we further evaluated the effect of the *DAND5* variant using a Nodal-dependent luciferase assay. The results showed a substantial decreased in the function of the DAND5 variant protein when compared to the wild-type DAND5 protein. Since p.R152H is localized in the functional domain of the DAND5 protein and because DAND5 inhibits NODAL by direct binding [31], we hypothesize that the protein alteration caused p.R152H variation might interfere with the interaction efficiency of DAND5-NODAL. Therefore, we further extrapolate that, most probably, during the embryonic development of patients with p.R152H DAND5, which has a DAND5 molecule with reduced activity, the overall activity of NODAL on the node is above the proper levels. This abnormal NODAL activity on the node might be reflected then in excess of NODAL activity on the left-lateral plate mesoderm and/or an ectopic expression of *NODAL* and *PITX2* on the right-lateral plate mesoderm of the embryo. Interestingly, the phenotypes observed in these two patients, are consistent with an ectopic expression of *NODAL* and *PITX2* on the right side of the embryo. Tetralogy of Fallot is a malformation of the cardiac outflow tract (OFT) that comprises ventricular septal defect, overriding of the aortic valve, right ventricular outflow tract obstruction (pulmonary stenosis or in its most severe form, pulmonary atresia), and right ventricular hypertrophy [33]. The OFT forms during heart looping as a result of a coordinated process uncompressing proliferation, differentiation, and migration of the pre-cardiac cells of the secondary heart field (SHF). These cells are known to be sensitive to the asymmetrically left expression of the *NODAL* and *PITX2* in the SHF [34]. Any imbalance in the LR patterning that might lead to an ectopic right-sided expression and loss-of-function of PITX2 results in reversed heart looping and abnormal shifting of the OFT [35, 36], suggesting that anomalous LR body patterning has the tendency to develop severe congenital heart defects. Moreover, cases of entirely bilateral expression of *NODAL* and *PITX2* in the cells of both left and right lateral plate mesoderm lead to left isomerism phenotypes, in which both atria present a left morphology, commonly associated with atrioventricular septal defects [37]. In agreement, it has been observed that the absence of Cerl2 (DAND5 mouse homolog) in *Cerl2* KO mice leads to several cardiovascular malformations, including incomplete atrial and ventricular septation, conotruncal defects, ventricular hypertrophy, and/or to laterality defects like left isomerism and thoracic heterotaxy or situs inversus [30, 31].

ExAC database describes DAND5 p.R152H as a SNV, detected in the heterozygous state in more than 1200 individuals, and present in 16 apparently normal homozygotes. Indeed, the mother of patient 2 is phenotypically normal but displays the DAND5 p.R152H variant in hererozygosity. Nevertheless, we cannot exclude the presence of a mild or undiagnosed heart defect on the mother of patient 2, or on the subjects of the genome projects, since detailed examination of these subjects is rarely available. In addition, the ExAC database and the American College of Medical Genetics and Genomics warns to the fact that in these population databases the presence of individuals with subclinical diseases among the supposed healthy individuals leading to minor discrepancies in some variant frequencies [32]. For example, in a study conducted in Newcastle, 30% of the patients (198/669 infants) were undiagnosed at the time of discharge from the hospital, and in 30/198 the diagnosis was only made after death [38]. Moreover, some phenotypes of CHD such as small atrial septal defects or ventricular defects may be asymptomatic and undetected throughout life [39]. Also, there are cases of apparently normal individuals whose laterality defects were only detected during adulthood [40]. Despite that, differences in the overall level of functional Nodal pathway might be explained by factors like differential allelic expressivity, an incomplete penetrance, the presence of genetic modifiers, and/or environmental factors. Interestingly, all *Cerl2* heterozygous mice are phenotypically normal, and ~40% of the *Cerl2* homozygous KO mice become normal adults, without any CHD and/or laterality defect [31]. Therefore, we are not in the presence of a Mendelian disorder. Moreover, the fact that 16 apparently normal homozygous individuals carry the DAND5 variant suggests incomplete penetrance and sustains the multifactorial etiology and complex trait of CHD. This indicates that the distribution and strength of the Cerl2 inhibitory signal, and ultimately the imbalance of the dose-sensitive Nodal signaling, might be sensitized by additional perturbations during LR patterning likewise it was observed in other genes of the NODAL signaling cascade [15, 17, 21]. For example, *cryptic* (*CFC1* mouse homolog) full-mutant mice presents randomization of abdominal situs and complex cardiac malformations such as atrial and septal defects, dextrocardia, DORV and TGA, but a normal configuration of the OFT was seen in one mutant [41]. In humans, it was identified and classified as disease variant due to its loss-of-function effect, a heterozygous single-base-pair deletion (G174 del1) in *CFC1* in two patients both with dextrocardia and TGA but one of the patients presented right isomerism, whereas the other patient presented left isomerism. This *CFC1* variant was also reported in one subject with sporadic DORV, ventricular septal defect, aortic arch hypoplasia [15] and in a patient with tetralogy of Fallot [17]. Interestingly, likewise we observed in p.R152H DAND5 variant, the mothers of these *CFC1* variant patients were found to carry the G174del1 alteration but none presented any clinical phenotype, suggesting incomplete penetrance. Accordingly, similar cases of reduced penetrance have been reported in CHD by others [25, 42].

Although the DAND5 p.R152H did not attest a specific genotype-phenotype link, our functional results support a model in which the proper levels of Nodal signaling, on both left- and right-lateral plate mesoderm, are particularly sensitive to gene dosage effects for all genes involved in the pathway. The imbalance in dosage-sensitive Nodal signaling is a final common way for laterality defects and associated CHDs.

## Conclusions

In conclusion, we report a missense heterozygous variant, in the *DAND5* gene of two unrelated Portuguese patients affected with congenital heart defects arising from possible left-right defects. Although the prediction analysis was inconclusive, the localization of the variant, the evolutionary conservation, and more importantly the functional assay suggest a possible role of this variant in the risk of disease. Our findings firstly prime DAND5 as one of multiple candidate determinants involved in the complex trait mechanism that causes CHDs, linking the human vulnerability for CHDs and the reduced activity of heterozygous DAND5 p.R152H variant. Nevertheless, an analysis of the DAND5 variant in a larger sample, to confirm its true CHD risk, should not be excluded.

## Additional files

Additional file 1: Figure S1. Luciferase assay with a dose response for the mutant protein versus the wild type protein. The results confirm the decrease in the DAND5 protein activity when compared the DAND5 variant with the wild-type DAND5 protein at three different concentrations. (TIFF 60 kb)

Additional file 2: Figure S2. Luciferase assay with a dose response for the mutant protein versus the wild type protein. The results confirm the decrease in the DAND5 protein activity when compared the DAND5 variant with the wild-type DAND5 protein at three different concentrations. (TIFF 60 kb)

Additional file 3: Figure S3. Western Blot of mouse wild-type and mouse variant DAND5/Cerl2 protein. Since the available antibodies against human DAND5/Cerl2 are not specific enough to detect the human protein, we performed a western blot assay using lysates of cells transfected with the mouse DAND5/Cerl2 WT and DAND5/Cerl2 variant proteins, and no difference was observed in the protein production. Curiously, the conditioned medium of the same cells showed a higher accumulation of the DAND5/Cerl2 variant protein when compared to the DAND5/Cerl2 WT protein. CM – conditioned media; Mut – Mutant protein; WT – wild-type protein. (TIFF 2995 kb)

## Abbreviations

1000GP: 1000 genome project
ARE: Activin responsive element
ASD: Atrial septal defects
AVSD: Atrioventricular septal defects
CHD: Congenital heart disease
DILV: Double inlet left ventricle
DORV: Double outlet right ventricle
ExAC: Exome aggregation consortium
HGM: Human gene mutation
L-LPM: Left-lateral plate mesoderm
LR: Left-right
OFT: Outflow tract
R-LPM: Right-lateral plate mesoderm
SHF: Secondary heart field
SNP: Single nucleotide polymorphism
SNV: Single nucleotide variation
TGA: Transposition of the great arteries
TOF: Tetralogy of fallot
VSD: Ventricular septal defects
