# Supplementary figures and images for: Functional study of DAND5 variant in patients with Congenital Heart Disease and laterality defects
Source: BMC Med Genet. 2017 Jul 24;18:77. doi: 10.1186/s12881-017-0444-1 (PMC5525210; doi:10.1186/s12881-017-0444-1)

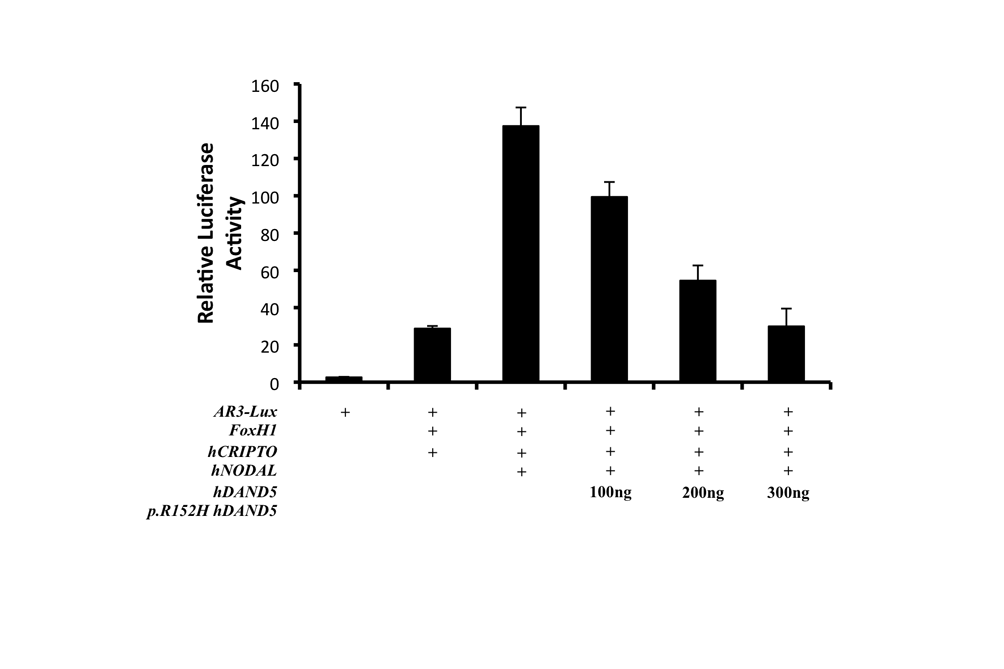

Supplement: Supplementary file 1 — Luciferase assay with a dose response for the mutant protein versus the wild type protein. The results confirm the decrease in the DAND5 protein activity when compared the DAND5 variant with the wild-type DAND5 protein at three different concentrations. (TIFF 60 kb) [file 12881_2017_444_MOESM1_ESM.tif]

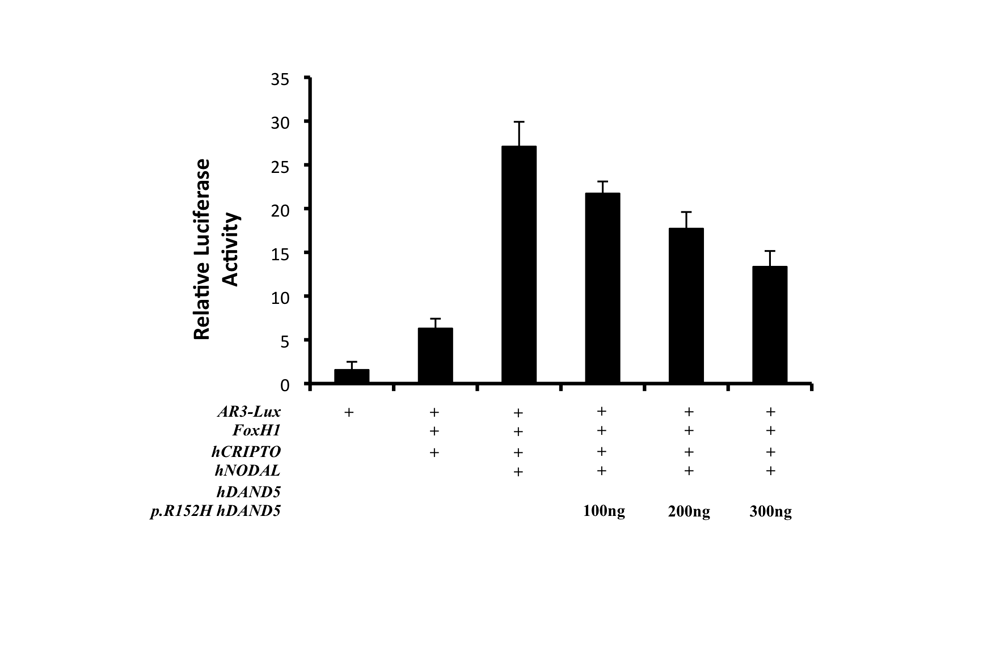

Supplement: Supplementary file 2 — Luciferase assay with a dose response for the mutant protein versus the wild type protein. The results confirm the decrease in the DAND5 protein activity when compared the DAND5 variant with the wild-type DAND5 protein at three different concentrations. (TIFF 60 kb) [file 12881_2017_444_MOESM2_ESM.tif]

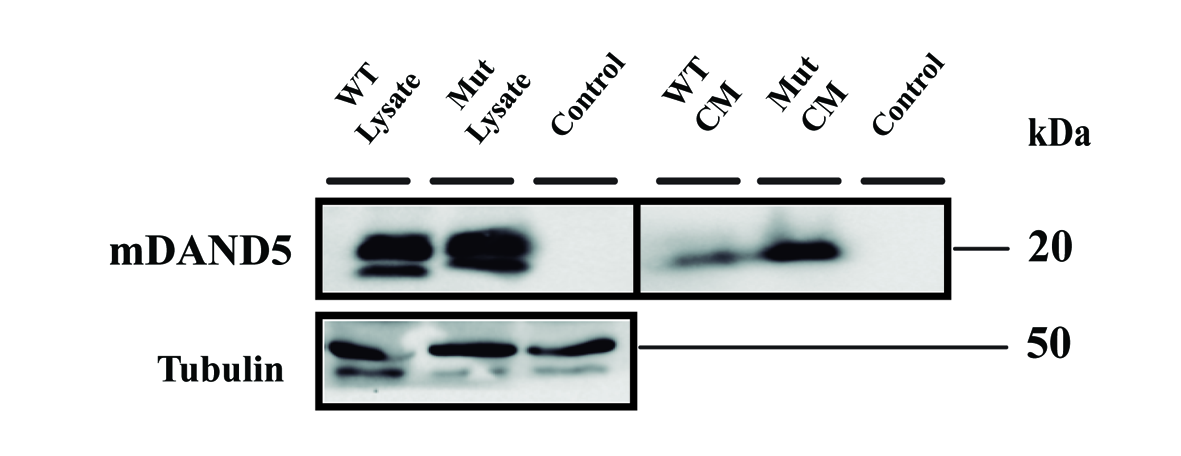

Supplement: Supplementary file 3 — Western Blot of mouse wild-type and mouse variant DAND5/Cerl2 protein. Since the available antibodies against human DAND5/Cerl2 are not specific enough to detect the human protein, we performed a western blot assay using lysates of cells transfected with the mouse DAND5/Cerl2 WT and DAND5/Cerl2 variant proteins, and no difference was observed in the protein production. Curiously, the conditioned medium of the same cells showed a higher accumulation of the DAND5/Cerl2 variant protein when compared to the DAND5/Cerl2 WT protein. CM – conditioned media; Mut – Mutant protein; WT – wild-type protein. (TIFF 2995 kb) [file 12881_2017_444_MOESM3_ESM.tif]
